# Integrated evaluation of groundwater hydrochemistry using multivariate statistics and irrigation-based water quality indices

**DOI:** 10.1038/s41598-025-09874-3

**Published:** 2025-07-10

**Authors:** Raisul Islam, Vinod Kumar Kushwah, Nakul Gupta, Ashish Kumar, Rajesh Goyal, Parveen Berwal, Faisal M. Alfaisal, Ali Majdi, Osamah J. Al-sareji, Majed Alsubih

**Affiliations:** 1 Civil Engineering Department, GLA University, Mathura, Uttar Pradesh 281406 India; 2 NICMAR Institute of Construction Management and Research, NICMAR, Delhi-NCR, Bahadurgarh, 124507 India; 3 Division of Research and Development, Lovely Professional University, Phagwara, India; 4 Civil Engineering Department, Galgotias College of Engineering and Technology, Greater Noida, 201310 India; 5 Department of Civil Engineering, College of Engineering, King Saud University, 11421 Riyadh, Saudi Arabia; 6 Department of Buildings and Construction Techniques Engineering, College of Engineering, Al-Mustaqbal University, Hillah, Babylon 51001 Iraq; 7 Sustainability Solutions Research Lab, Faculty of Engineering, University of Pannonia, Egyetem Str. 10, Veszprém, 8200 Hungary; 8 Civil Engineering Department, College of Engineering, King Khalid University, 61421 Abha, Saudi Arabia

**Keywords:** Groundwater analysis, Hydrochemical facies, Multivariate statistical techniques, USSL, Water quality index, Wilcox, Civil engineering, Environmental sciences

## Abstract

This research evaluates the appropriateness of groundwater for potable and irrigation need in the Mathura District of Uttar Pradesh, India. Fifteen different hydro chemical parameters selected for analysis, they are pH, total dissolved solids (TDS), electrical conductivity (EC), Total Hardness (TH), Mg^2+^, Cl^−^, Ca^2+^, Na^+^, K^+^, NO_3_^−^, SO_4_^2−^, PO_4_^3−^, F^−^, CO_3_^2−^, and HCO_3_^−^ In the present research various approaches such as Multivariate statistical techniques (MSTs), Arithmetic water quality index (WQI), sodium absorption ratio (SAR), permeability index (PI), sodium percentage (Na%), US salinity, were examined. The WQI assessment reveals that 65% poor quality, 5% very poor quality and 15% water is not suitable for drinking purpose category. Thus, it indicates that the majority of groundwater surpassed acceptable thresholds for potable water consumption. Additional indices indicate that several metrics surpass their acceptable limits, rendering most samples inappropriate for irrigation. CaMgCl, NaCl and CaNaHCO_3_ attributed to interfaces between water and rock and ion exchange processes in Sodium–Potassium from water and Calcium-Magnesium from rock. Furthermore, the US Salinity graphic confirms that the majority of groundwater samples demonstrate very high salinity risks and Sodium hazard, especially for elevated salt concentrations. Influence the soil fertility, permeability of soil and crop growth. The findings of this study will be beneficial to policymakers and decision-making authorities in executing sustainable water quality initiatives and efficient management of water resources as per scientific principles of various global and national agencies.

**Supplementary Information:**

The online version contains supplementary material available at 10.1038/s41598-025-09874-3.

## Introduction

Water is one of the most important most important resource required to support living organism^1^. Accessibility to sources of freshwater is depliting due to human interference and growing human population. The conservation of water resources is the main concern of human beings^2^. Groundwater is an essential resource for both drinking and irrigation applications^3–5^. Globally, humans utilize around 65.5% of groundwater for drinking, 19.5% for irrigation, and 15% for industrial operations. In the meantime, a number of recent droughts and overuse of groundwater are lowering groundwater levels and degrading its quality^6^. One of the most severe problems of developing countries is the improper management of huge amounts of waste generated by various anthropogenic activities^7^. In India, groundwater used as the primary water source for both drinking and farming activities. Analysis of groundwater quality provides valuable insights into the geological rock formations and indicates the processes of groundwater recharge, discharge, and storage. Geological formations and human activities have a significant impact on the physical and chemical parameters that determine the variation in groundwater quality in a given geography. Mathura is a historic, cultural and religious city located in the northern Indian state of Uttar Pradesh. Mathura is facing issues of declining groundwater quality and scarcity. The ground water of Mathura is saline and hard water. Limited research are available on ground water quality of Mathura district. So, that this research study provided the valuable as basic information for future aspects and research. Rapid development of Mathura causes enhanced need for groundwater resources. Owing to the rapid urbanization, industrialization, and population increase, a variety of by-products are produced from day-to-day living and are frequently disposed of carelessly, creating serious environmental issues^8^. Mathura groundwater has been identified as a major source of irrigation and drinking water, one of the most important aspects of managing water resources is to check the water quality and regular monitoring. The primary factors that can influence the chemical quality of groundwater are precipitation levels, leaching of surface and agriculture run off, the geological configuration of the area, urbanization, seepage of domestic waste, surface runoff, and the mineral composition of the aquifer resource^9–11^. The proficient planning of water resources, irrigation, and soil conservation, to address the problems related to the management of water and land resources^12^. Monitoring programmes aim to incorporate regular collection of groundwater samples from multiple predetermined locations in order to provide an accurate representation of the groundwater quality in the specific area being studied. Recent research findings established the utility of Multivariate statistical techniques, which have been employed for effectively determine and assess the hydrochemical characteristics of aquifers^13–16^. MST (Multivariate Statistical Techniques) effectively elucidate the aquifer’s attributes and behavior regarding contamination of groundwater. The utilization of various MSTs, including cluster analysis (CA) and discriminant analysis (DA), has garnered significant interest in water quality research, facilitating the interpretation of complex information structures to enhance understanding of the quality of water. CA was utilized to examine the spatial distribution of the sampling sites. This type of approach is a standard technique for classifying characteristics into groupings^17^. CA is typically endorsed by DA for evaluation and frequently referred to as pattern exploration tools^18^. Hamma et al. (2024) conducted a comprehensive hydrochemical assessment of groundwater in the Ain Sefra region of southwestern Algeria. The analysis identified four distinct groups of groundwater based on their chemical composition using hierarchical cluster analysis and several processes significantly influence groundwater chemistry, including: Water-rock interaction, Reverse ion exchange, anthropogenic pollution and only 2.32% of the samples deemed unsuitable^19^. DA to evaluate the spatial discrepancies and the analysis of the water quality information sets. Multiple indexing methods exist that integrate water quality characteristics with the quality status expressed as a single numerical value. An essential water quality indicator, such as the arithmetic WQI, simplifies the representation of water quality and enhances its understanding^20,21^. Jodhani et al. (2025) assessed the Water Quality Index (WQI) of groundwater in Valsad District of southern Gujarat. The study results indicated that the WQI varied from 14.20 to 41.98, implying that groundwater in the Valsad district is appropriate for consumption. The piper diagram revealed that the Ca^2+^ was the dominating cation, followed by K^+^, Na^+^, and Mg^2+^. Among the anions, HCO_3_^−^ had the highest concentrations, succeeded by SO4^2−^, NO_3_^−^, and Cl^−^^22^. Gautam et al. 2024 The study on groundwater quality characterization in the Jakham River Basin in Rajasthan utilized an integrated approach combining a Water Quality Index and multivariate statistical techniques. The study found that during the pre-monsoon and post-monsoon season 63.42% and 42.02% of groundwater samples respectively were classified as ‘good’, indicating they are suitable for human consumption^23^. Understanding hydrochemistry and chemical composition is crucial for evaluating groundwater quality and its appropriateness for diverse applications^24,25^. The approaches suggested by Piper and chloro-alkaline indices (CAI-1 & 2) have been employed to systematically examine the hydrochemical properties and ion exchange mechanisms in the rock-water interactions of the groundwater in Mathura City. The appropriateness of groundwater for irrigation operations is measured by several indicators such as sodium absorption ratio (SAR), USSL diagram, sodium percentage (Na%), and Wilcox diagram^23^. The groundwater quality was evaluated using the WQI as well as the SAR, Na%, USSL and piper diagram to evaluate its suitability for portable and irrigation uses. These indices and graphical representations are especially beneficial for enlightening peoples and pertinent agencies regarding the groundwater quality across various sustainable management of water regimes. In this study, a comprehensive technique for evaluating groundwater quality in the Mathura region is presented. This methodology integrates multivariate statistical approaches with water quality and irrigation indices, which is an approach that has not been applied in this location before. Despite the fact that the Mathura region is experiencing an increase in industrial and agricultural development, there is a lack of comprehensive and systematic examinations of the quality of the groundwater. That deficiency is addressed by this study, which offers the first comprehensive hydrochemical assessment that has been particularly created for this region. This study present an example of a novel feature by incorporating the Water Quality Index (WQI) for the evaluation of potable water and the Irrigation Indices. The objective of this study is to determine the hydrochemical properties and appropriateness for potable and irrigation needs of groundwater in the Mathura region. The findings proposed in the paper have substantial implications for the development of the field through improved applications. The research contributes to advancing knowledge and practical applications. In addition to this, topic is timely and relevant, addressing issues that are currently of great interest to researchers, policymakers and to the public.

## Materials and methods

### Study area

**Fig. 1:**
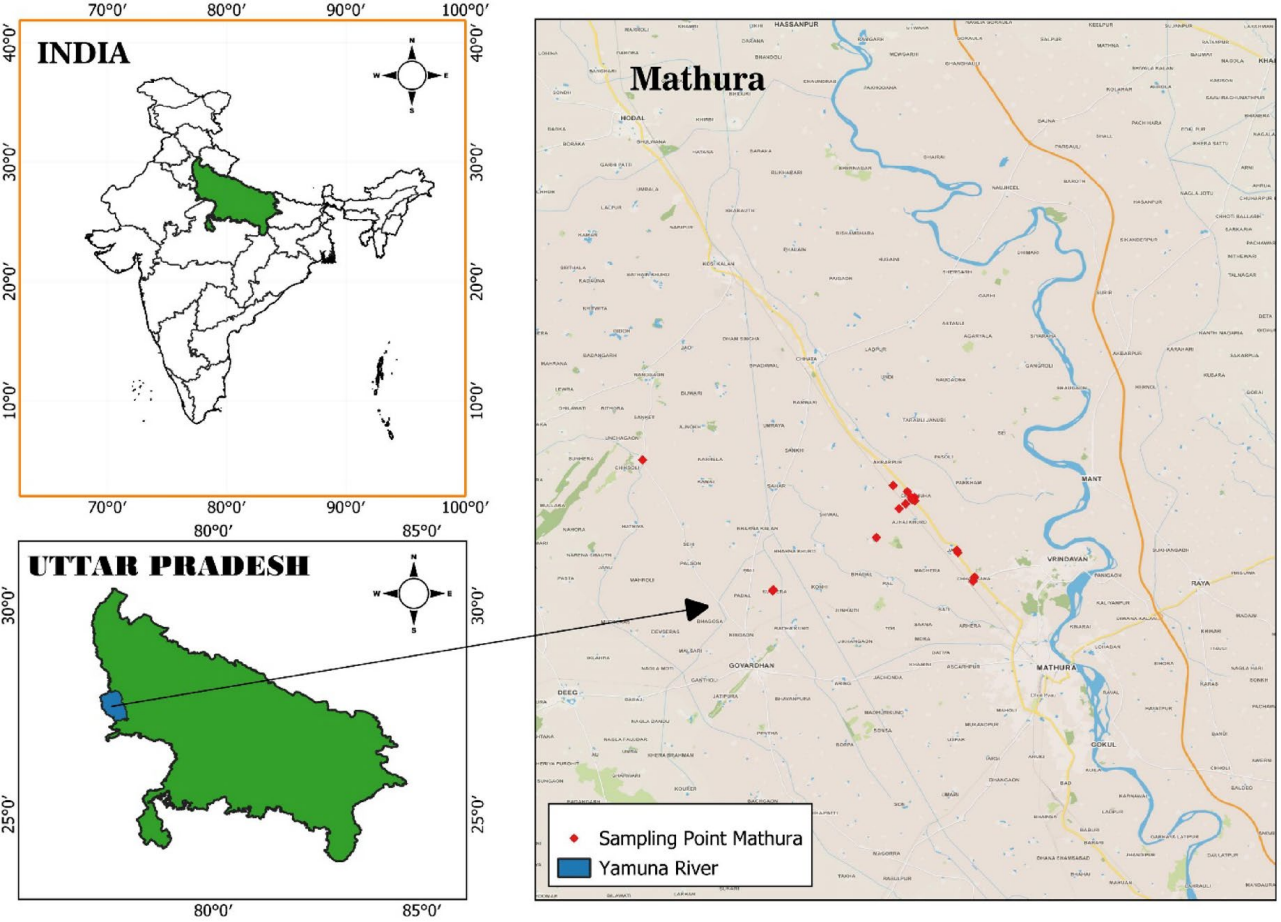
Study area map showing sampling locations.

Mathura is situated around 50 km north of Agra and 150 km south of Delhi. The district is situated between latitudes 27°15′ N and 27°58′ N, and longitudes 77°16′ E and 77°57′ E. According to the 2011 census, the district has an approximate population of approximately 2.543 million. The population density is 770 individuals per square kilometer. The mean annual precipitation is 625 mm. The climate is characterized as subtropical humid, featuring a hot, arid summer and a mild, cool season. Approximately 88% of precipitation occurs between June and September. The average daily lowest temperature is approximately 8 °C, while May is the month with the highest temperatures, featuring an average daily maximum temperature of 44 °C and an average daily minimum temperature of 25 °C. With the arrival of the monsoon, daytime temperatures decrease significantly. The district’s relative humidity fluctuates monthly, reaching a maximum of 82–91% in July and August because of monsoon precipitation. The prevailing winds are typically low to moderate, with an average velocity of 5.8 km/hr. The possibility of evapotranspiration measures 1467.2 mm. The soil types of the district, which formed by the Indo-Gangetic alluvium and the exhibit significant diversity across various regions. Figure 1: Study area map showing sampling locations.

### Sample collection and analysis of samples

Representative groundwater samples were obtained from a total of 20 specifically chosen sites within the Mathura district. The water samples were mostly taken from handpumps in the year, 2024 using the established protocols of the American Public Health Association (APHA), 2012. Prior to sampling, discharge from the hand pump was kept operational for a duration of 4–5 min in order to remove any stagnant water present in the pipes. The collection of every sample was conducted using a 1000-mL sterile high-density polypropylene bottle. Three samples were collected from different sampling site. The sampling site and the source were geocoded using a portable global positioning system to determine their geographic coordinates. The pH, total dissolved solids (TDS), and electrical conductivity (EC) were determined immediately onsite using portable testing instruments. Once collection, the samples were immediately placed in an icebox to maintain their integrity and prevent any deterioration. They were then carefully transported to the laboratory and subsequently stored at 4 °C to assure their preservation and availability for further analysis. The relative ranges of the Total Hardness (TH), Mg^2+^, Cl^−^ and Total Alkalinity were measured using the volumetric titration technique, while Ca^2+^, Na^+^ and K^+^ were evaluated using the flame photometer method. The primary anions NO_3_^−^, SO_4_^2−^, PO_4_^3−^ and F^−^ were measured using a spectrophotometer following the methodology outlined in the APHA, 2012. The remaining anions, CO_3_^2−^, and HCO_3_^−^, were identified by titrimetric methods.

## Materials and methods

### Multivariate statistical techniques (MSTs)

Due to the fact that each sampling site is distinguished by a significant number of physical as well as chemical variables, a regional hydrogeochemical study is problem which involves multiple variables. The primary determinants of groundwater quality were analyzed using multivariate statistical techniques. The analytical data analysis was processed using IBM SPSS version 20.0 software. Initial statistical measures, including multivariate evaluations, were computed for the individual variables. MSTs are used to evaluate the analytical data in order to locate the sources of pollutants. MSTs provides a quantitative and independent method of groundwater classification that enables the grouping of groundwater samples and the establishing of correlations between chemical characteristics and groundwater samples.

CA and DA are two examples of multivariate statistical techniques that are frequently utilized in environmental research^27^. Cluster analysis (CA) is one of the multivariate methods that categorizes the objects according to the characteristics they possess. In accordance with a predetermined set of selection criteria, it organizes the objects in such a way that each individual object in the cluster is identical to the others in the cluster. Clusters that are generated in this manner display both high levels of internal homogeneity and substantial variability between classes^28^. The approach that is utilized the most frequently is known as hierarchical agglomerative clustering (HCA)^29^. The database was evaluated using Ward’s method of linkage, with Euclidean distance serving as the measure of similarity. This application took place after the data were scaled using z-transformation. A dendrogram, also known as a tree diagram, is used to represent it^30^.

DA is a technique for supervised arrangement recognition that is applied for the purpose of classifying objects or cases into exhaustive and mutually exclusive clusters. This classification is accomplished by using a set of independent variables as the basis for the classification. In situations where the variable that is dependent is a variable with a category and the variables that constitute the independent variable are metric, this statistical technique is an appropriate choice^31^. As shown in Eq. (1), the discriminant function (DF) for each classification:

1 $${\text{f}}\left( {{\text{G}}_{{\text{i}}} } \right) = {\text{k}}_{{\text{i}}} + {\text{}}\mathop \sum \limits_{{{\text{j}} = 1}}^{{\text{n}}} {\text{w}}_{{{\text{ij}}}} {\text{P}}_{{{\text{ij}}}} {\text{}}$$

Where i represents the no. of groups (G),

k_i_ represents the constant that is inherent to every group,

n represents the number of variables that are used to classify a set of information into a particular group, and w_j_ represents the weight coefficient that is assigned by DA to a particular variable that has been selected (p_j_).

### Hydrochemical groundwater mechanism

Hydrochemical investigations were conducted to characterize the groundwater conditions, identify a geographic distribution of chemical variables, and illustrate the physiochemical processes that impact the chemical composition of groundwater using various hydrochemical graphs such as piper diagram^32,33^. An evaluation of the quality of groundwater for drinking as well as irrigation applications was carried out in accordance with the standards established by the Bureau of Indian Standards (BIS) in 2012 and the World Health Organization (WHO)^34,35^. As a result of the consequences of weathering and anthropogenic components, the overall quality of groundwater has been drastically impacted.

The Piper diagram is a computational tool applied for deducing hydro-geochemical facies. The piper plots consist of two triangles, one designated for graphing major cations and the other being used for plotting major anions. An amalgamation of the cations and anion domains results in a singular point within a diamond-shaped field, enabling the derivation of inferences based on the hydro-geochemical aspects concept.

### Evaluation of chloro alkaline ions mechanism

A comprehensive understanding of the ion exchange between underground water and its host environment throughout residence or travel can be achieved by the analysis of chloro-alkaline indices. Schoeller, 1977 proposed two chloro alkaline indices, CAI-1 and CAI-2, to measure the transfer of ions between aquifers and its surrounding environment^36^. CAI is negative when there is a charge exchange between sodium and potassium in water with calcium and magnesium in rocks. CAI is positive when there is no base exchange.

These chloro-alkaline indices are calculated using mathematical Eqs. (2) and (3):

2 $$CAI - 1 = \frac{{Cl^{{ - }} - Na^{ + } + K^{ + } }}{{Cl^{ - } }}$$

3 $$CAI - 2 = \frac{{Cl^{{ - }} - (Na^{ + } + K^{ + } )}}{{\left( {SO_{4}^{{2 - }} + HCO_{3}^{ - } + CO_{3}^{{2 - }} + NO_{3}^{ - } } \right)}}$$

All quantities are evaluated in milli equivalent per litre (meq/L).

### Classification of groundwater quality for irrigation application

In order to determine groundwater is suitable or not for irrigation, it is essential to compare the amount of sodium present in the system to the total number of electrolytes present. The presence of high sodium in water makes it unsuitable for irrigation activities. This is due to the fact that sodium causes ion exchange processes, which have the tendency to affect the capacity of soils to sustain crop production. The sodium ion accumulates in cation exchange locations, which results in the dispersion of soil aggregates and a subsequent reduction in the permeability of the soil^37^.

Assessment of the irrigational appropriateness of groundwater in the investigated area was conducted using SAR, EC, USSL, Na%, and Wilcox diagram. These methods were selected based on a comprehensive understanding of the chemistry of groundwater and its appropriateness for agricultural applications. Typically, the water loss through evaporation and the decrease in groundwater levels, especially during the hot season, increase the concentrations of chemical constituents in the groundwater, which impacts both the soil and the plants^38^. This issue is paramount in the majority of semi-arid regions characterized by rock dominance. The primary concern is the elevated sodium content in water, leading to the development of alkaline soil, and substantial salt concentration, resulting in the formation of saline soil^39^. The mineral make-up of irrigation water has a significant impact on the amount of crop yield. It is possible that crop yield could be hindered by the existence of an excessive quantity of dissolved Ions in water used for irrigation.

According to Wilcox diagram, groundwater for irrigation purposes was classified according to the percentage of sodium and the electrical conductivity of the water^40^. Within the context of determining whether water is suitable for irrigation or not. Using the implementation of specific methodology, the US Salinity Laboratory of the Department of Agriculture (USSL) has been able to provide an explanation for the appropriateness of water for agricultural purposes^41^. In order to assess the suitability of water for irrigation, the USSL categorization is a graphical representation used to assess its chemical structure, namely its salinity risk (electrical conductivity) and sodium concentration (Sodium Adsorption Ratio). The USSL graph categorizes samples of water into four primary groups: C1 (Low), C2 (Medium), C3 (High), and C4 (Very high) based on Salinity hazard. The SAR consists of four categories: S1 (Low), S2 (Medium), S3 (High), and S4 (Very high).

### Percent sodium (%Na)

Percent Sodium is one of the indices that is frequently used to determine whether or not groundwater is suitable for irrigation purposes is the percentage of sodium ions. Wilcox (1955) demonstrated that the percentage of sodium is a fairly standard measurement that is utilized to a significant degree in order to determine the quality of water that is intended for use in irrigation.

An excessive concentration of sodium ions has an effect on the permeability of the soil, which in turn has an effect on the growth of crops^42^.The combination of sodium and carbonate can result in the formation of alkaline soils, whereas the combination of sodium and chloride would result in the formation of saline soils. There is no evidence that either of these soils promotes the development of plants^43^. The amount of sodium that is present in irrigation waters is typically expressed as a percentage of sodium, which is determined by the Eq. (4).

4 $${\text{\% Na}} = \frac{{({\text{Na}}^{ + } + {\text{K}}^{ + } )}}{{\left( {{\text{Ca}}^{{2 + }} + {\text{Mg}}^{{2 + }} + {\text{Na}}^{ + } + {\text{K}}^{ + } } \right)}} \times 100{\text{}}$$

Where, all quantities are evaluated in millie-equivalents per litre.

### Sodium adsorption ratio (SAR)

In the evaluation of irrigation water, the SAR, which is a numerical proportion of sodium ions to calcium ions and magnesium ions in the water sample, is the most commonly used metric. The SAR parameter, which was developed by Richards in 1954, is utilized in water used for irrigation to refer to the capacity of the soil to discharge Ca^2+^ and Mg^2+^ and to accumulate Na^+^ from groundwater. This results in a decrease in the porosity of the soil. In the USSL graphical diagram of irrigation water, the SAR values versus the EC values for groundwater samples taken from the area under study were plotted accordingly. To estimate SAR, the formula that follows Eq. (5) can be utilized^44^.

5 $$\:SAR=\:\frac{{\text{N}\text{a}}^{+}}{\sqrt{\left({\text{C}\text{a}}^{2+}+{\text{M}\text{g}}^{2+}\right)/2}}$$

### Water quality index (WQI)

WQI is a basic but significant mathematical tool for evaluating the overall condition of groundwater based on various criteria that assess its appropriateness for drinking or irrigation needs^45^. A Water Quality Index (WQI) is a singular numerical value that conveys the overall status of the water quality at a specific time and place. The primary objective of WQI is to convert intricate quality of water statistics into data that is comprehensible and applicable to the general population^46^. The weighted arithmetic WQI method was employed to accurately assess the appropriateness of groundwater for potable use. A quantity rating scale (Qj) for every parameter was calculated by dividing its recommended concentration as specified by BIS and WHO guidelines Eq. (6).

6 $$\:WQI=\sum\limits_{j=1}^{n}{Q}_{j}{W}_{j}$$

Location Q_j_ = sub index for the jth quality of water parameter, W_j_ = weight attributed to the jth quality of water parameter, n = quantity of quality of water parameters.

According to the above information, the Water Quality Index categorizes drinking water into five classifications: excellent quality, good quality, poor quality, very poor quality, and unsafe as illustrated in the Table 9^47^.

## Result and discussion

### Assessment of hydrochemistry of groundwater

Assessment of water quality can provide insights into the various environmental conditions. This study presents the results of the analysis for all the hydrochemical quality parameters including basic statistics of the respective values of groundwater samples of the study area in Table 1. The statistical analysis of groundwater samples in study area, yielded the following parameters: minimum, maximum, mean, and standard deviation values. Chemical composition of overall groundwater samples from the study area exhibits significant variation.

**Table 1:** Summary of descriptive analysis of groundwater quality parameters.

Parameters	No. of Sites	Minimum	Maximum	Mean	^1^Std. error	^1^Std. Dev	Skewness	Kurtosis
pH	20	6.36	8.79	7.4445	0.167	0.746	0.435	− 0.872
EC	20	1118	9160	4021.80	566.92	2535.35	0.806	− 0.626
Tur	20	1.00	14.00	3.1500	0.685	3.065	2.778	8.373
TDS	20	962	6647	2696.50	388.33	1736.70	0.939	− 0.222
TH	20	70	2240	1011.45	141.33	632.06	0.575	− 0.481
TA	20	60	740	414.20	37.76	168.89	− 0.252	− 0.005
Ca^2+^	20	12	525	210.75	31.46	140.69	0.723	− 0.120
Mg^2+^	20	9.70	442	143.41	26.002	116.28	1.386	1.519
Na^+^	20	190	1220	562.50	79.071	353.61	0.471	-1.356
K^+^	20	0.00	95	16.05	6.756	30.21	2.139	2.994
HCO_3_^−^	20	36.60	451	252.66	23.037	103.02	− 0.252	− 0.005
CO_3_^2−^	20	23.40	288	161.53	14.728	65.86	− 0.252	− 0.005
Cl^−^	20	106	2154	726.75	149.747	669.69	0.902	− 0.693
SO_4_^2−^	20	75	1640	496.72	121.162	541.85	1.320	0.321
F^−^	20	0.26	0.97	0.63	0.049	0.222	− 0.131	-1.288
NO_3_^−^	20	0.76	35.5	11.05	2.363	10.57	0.85	− 0.363
PO_4_^3−^	20	0.1	0.5	0.205	0.029	0.131	0.97	− 0.34

pH is the universally used term to quantify the degree of acidity or alkalinity. The pH of groundwater is a crucial parameter for assessing its quality and is regulated by the concentration of dissolved carbon dioxide, carbonate, and bicarbonate^48^. The pH measurements of the groundwater samples obtained from the examination area ranged from 6.36 to 8.79, with an average value of 7.44. This result indicate that the groundwater has a bit of an acidic but slightly alkaline characteristic. The pH values of all water samples from the sampling sites were found to be within the permitted range according to the BIS, 2012 standards. pH, while not strictly affecting human health, shows an influence on all biological and chemical processes^49^. EC is widely utilized as the parameter for evaluating water salinity. Electrochemical conductivity (EC) ranges from 1118 to 9160 µS/cm, with an average value of 4021.80 µS/cm. The World Health Organization (2017) specified that the highest allowable concentration of EC in drinking water is 1,500 µS/cm at 25 °C^35^. 19 sampling sites of the groundwater of EC surpasses the allowable threshold except at sampling location of 12. Higher EC values in the investigated area imply an increase in salt concentration in the aquifer system. In the study of S. Ahmed et al. (2020) during 2016, the average concentration of EC as observed that most of samples were lies on above permissible limit of prescribed by WHO^50^.

Deposition of suspended particles in the water results in a turbid or muddy visual effect. The observed phenomenon could be attributed to erosion and accumulation of iron occurring within the handpumps pipe, which is the primary origin of elevated levels of suspended matter in water. The measured turbidity concentrations varied between 1 and 14 NTU with mean of 3.15 NTU. 15% sampling sites of groundwater fell above the permissible threshold recommended by BIS, 2012. Total Dissolved Solids (TDS) is the primary measure of the total mineral content and salinity range in groundwater^51^. TDS concentrations ranged from 962 to 6647 mg/L with average level of 2696.50 mg/L, indicating 45% of groundwater sampling locations falling within the allowable level of 2,000 mg/L as per BIS 2012. The highest TDS observe was found at sampling site 12, while the lowest was at groundwater sampling site 5. The elevated level of TDS in the groundwater sampling sites are caused by the influence of rock-water relationship on the recharge hydrology, domestic sewage processes, salt dissolution with rainfall infiltrations and soil leaching^52^. The main factor contributing to water hardness is the existence of cations, such as calcium and magnesium, as well as anions, including carbonate, bicarbonate, chloride, and sulphate, in water^53^.

Total Hardness of groundwater ranged from 70 to 2240 mg/L with mean value of 1011.45 mg/L. 14 sampling sites were found above the permissible limit of groundwater as per BIS, 2012. The water hardness is caused by the existence of alkaline earth elements, namely calcium and magnesium. TA of water is a quantitative assessment of its capacity to mitigate the effects of acids. This parameter mainly indicates the levels of HCO_3_^+^, CO_3_^2−^, and OH^−^ present in the water. TA concentrations ranged from 70 to 740 mg/L, with an average concentration of 414.20 mg/L, 90% of sampling sites of groundwater falls well within the permitted limit of 600 mg/l according to the BIS, 2012. The primary source of alkalinity in natural water is the dissolution of CO_2_ in the water mixture. In the study of Singh et al. (2012) during 2010, the average concentration of TA lies in 150 to 1000 mg/L^54^. Naturally occurring water often contains a high concentration of both magnesium and calcium ions. The groundwater samples exhibit a calcium concentration ranging from 12 to 525 mg/L with mean of 210.75 mg/L. A maximum Ca^2+^ concentration of 66.4 is observed at site 6 within the study area. 45% of water samples fall above the permissible level of 200 mg/L (BIS, 2012). Calcium may have been increase through the process of carbonate dissolution in the chemistry of groundwater. The concentration of magnesium ions varies from 9.70 to 442 mg/L. The maximum concentration was found 442 mg/L at sampling site 20. Dolomite disintegration and silicate weathering are two prevalent origins of Mg^2+^. Naturally occurring sodium ions commonly enter water through processes such as decay and weathering of minerals and rock formations.

The sodium concentration in the ground water samples varied from 190 to 1220 mg/L. Most of the sampling sites of groundwater fall above the permissible limit of sodium as per BIS, 2012 and WHO, 2011^34,35^. The high concentration of sodium water is not suitable for irrigation activities. Elevated levels of sodium in drinking water can affect flavor and pose a risk for individuals with specific health conditions, such as hypertension, and can result in cardiovascular diseases, renal infections, high blood pressure, and hyperosmolarity. Potassium ions are introduced into water through natural processes such as soil intrusion and mineral dissolution. The element potassium is naturally occurring in minerals such as potassium chloride and potassium sulfate, and can be found in soil and natural water sources. The mean value of potassium in groundwater is 16.05 mg/L, while the concentration ranged of potassium in groundwater is 0.0 and 95 mg/l. 85% of sampling sites of water fall below the permissible limit of potassium. The present research observes that the level of K^+^ in groundwater is generally low related to other significant cations, mostly because of its small mobility. The decreased potassium concentration is ascribed to the increased capability of potash feldspar to withstand weathering caused by chemicals^55^.

Bicarbonate concentration ranges from 36.60 to 451 mg/L with an average concentration of 252.66 mg/L. Ground water sampling site of 4 and 16 exhibit the greatest concentration.

The presence of elevated bicarbonate (HCO_3_^−^) levels in the groundwater suggests the current state of mineral dissolution within the groundwater system. The solute carbon dioxide, cations, and other dissolved salts are independent factors that determine the concentration of carbonates in natural waters. The concentration of carbonate in ground water is ranged from 23.40to 288 mg/L with mean value of 161.53 mg/L. The elevated levels of chloride in the groundwater of the research area is attributed to the infiltration of wastewater from sewers, septic tanks, and industrial waste. The chloride concentration ranges from 106 to 2154 mg/L, yielding an average of 726.75 mg/L. The findings indicate that 30% of the groundwater samples in the study area fall above permissible limit set by the BIS, 2012. Chloride is the predominant naturally occurring form of chlorine and demonstrates exceptional ability to remain stable in water. SO_4_^2−^ is a prominent anion observed in natural water sources. The concentration of sulfate SO_4_^2−^ ranged from 75 to 1640 mg/L, with a mean value of 496.72 mg/L. 45% of sampling sites of groundwater in the study area meet the recommended limit of 200 mg/L set by the BIS, 2012. 35% of samples fall above permissible limit of sulphate. Sulphate (SO₄²^−^) is a prevalent anion present in natural water systems. Naturally occurring sulphates are found in minerals such as gypsum and anhydrite. Their release into water can occur via the process of mineral weathering. Tiwari et al. 2024 conducted a qualitative assessment of ground water in Sohagpur coalfield, Madhya Pradesh. The results reveals that the significant number of groundwater investigations had concentrations of TH, TDS, turbidity, and SO42 − that beyond the permitted limits set by the Bureau of Indian Standards (BIS), indicating that the water is unfit for direct consumption^56^.

Nitrate occurs naturally in soil and water due to the nitrogen cycle, in which nitrogen compounds are transformed into nitrate by processes such as nitrification. The soluble nitrate concentration varied between 0.76 and 35.5 mg/L, with an average of 11.05 mg/L. Nitrates are frequently employed in synthetic fertilizers to augment the growth of plants. Runoff originating from agricultural fields has the potential to transport surplus nitrates into water bodies. Nitrate is a constituent of sewage and has the potential to migrate into water bodies via wastewater discharges if not adequately treated. All 20 sampling locations of groundwater samples recorded nitrate concentrations below the acceptable threshold of 45 mg/l according to the Bureau of Indian Standards 2012. Nitrate in high concentrations is toxic and has the potential to cause various diseases, including blue baby disease and methaemoglobinemia in children, as well as gastrointestinal carcinomas^57^. Singh et al. (2024) identified increased nitrate concentrations in aquifers near Mathura region, attributing these to excessive fertilizer application and inadequate sanitary infrastructure^58^. Naturally occurring phosphate is found in minerals like apatite, which gradually weather to liberate phosphate ions into the soil and water. A range of 0.1 to 0.5 mg/L with mean of 0.205 mg/L was observed for phosphate level, with 80% of the samples falling within the acceptable threshold of 0.3 mg/l. Phosphates are frequently employed in the water samples due to fertilizers to augment the growth of crop plants. Hydrological runoff from agricultural fields can transport surplus phosphates into aquatic environments. Phosphate is constituent of sewage and can exacerbate nutrient contamination if not adequately processed. Taking into account the mean concentrations of cations and anions, the following is the order in which they are most prevalent: Na^+^ > Ca^2+^ > Mg^2+^ > K^+^ and Cl^−^ > SO_4_^2−^ > HCO_3_^−^ > CO_3_^2−^ > NO_3_^−^ > F^−^ > PO_4_^3−^.

### Computation of groundwater characteristics using hydrochemical facies

**Fig. 2:**
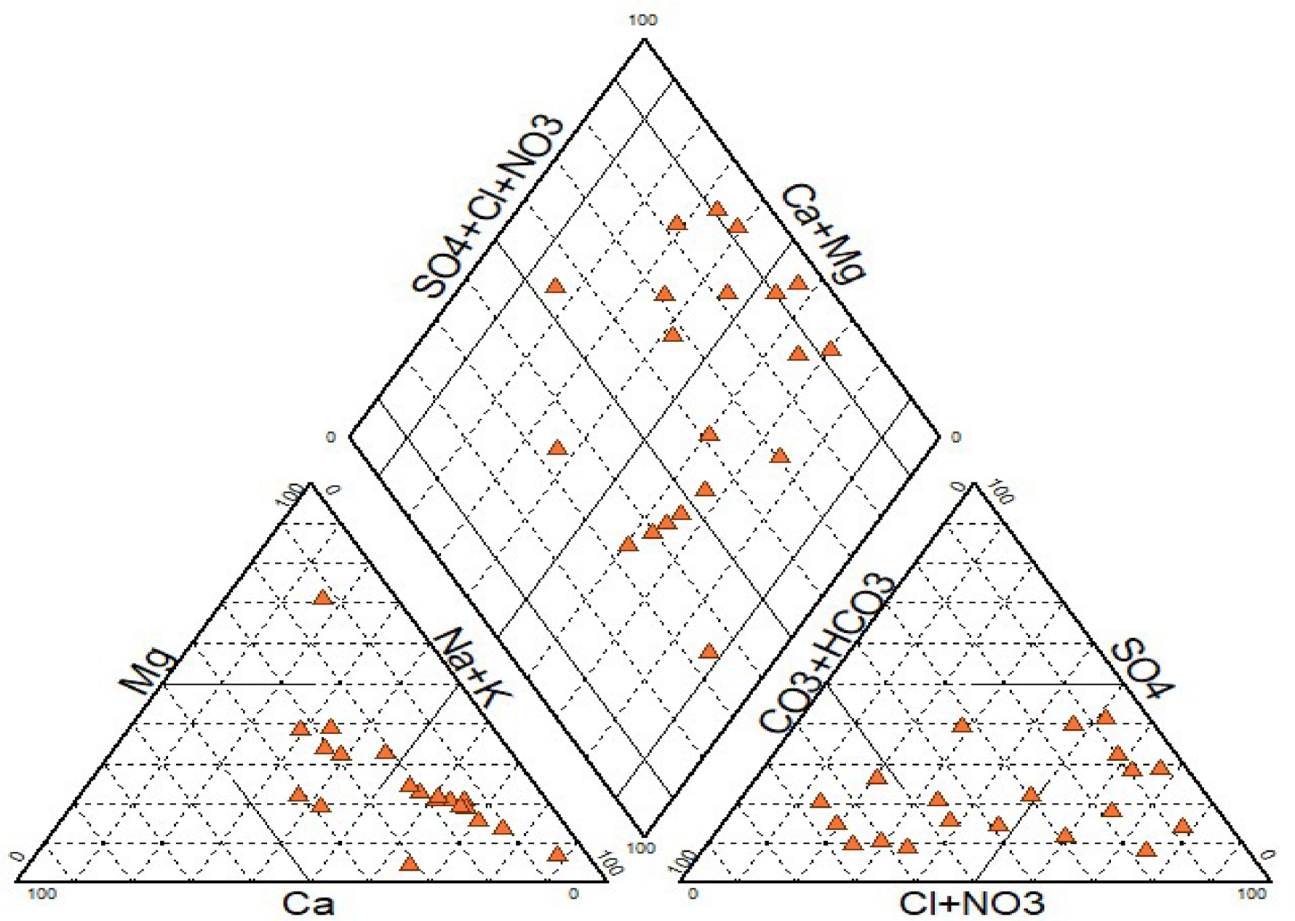
Piper plot showing hydrochemical facies of groundwater quality of study area.

The hydrochemical facies approach was formulated to comprehend and classify the mineral composition of water in various categories. Piper trilinear diagrams (1944) are valuable for elucidating chemical interactions among samples of groundwater more precisely than other plotting techniques. Plotting chemical characteristics of samples representative from the study area on a Piper trilinear diagram as illustrated in Fig. 2. The diagram comprises three clearly different fields, namely two triangular fields and one diamond-shaped field. Distinct groundwater types can be distinguished by their location within the diamond field. This diagram provides valuable characteristics and correlations for extensive sample groups^59^. Specifically, in the research region, The Piper diagram indicates that CaMgCl, NaCl and CaNaHCO_3_ are predominant water forms. The triangular plots of cation and anion concentrations make it abundantly clear that the Na type and the HCO_3_^−^ type and Cl^−^ type of water predominated, respectively. The chemical composition mostly arises from the process of mineral dissolution in the soil and the rocks it has come into contact with, at present or in the past. Additionally, it has been suggested that the interaction between rock and water due to silicate weathering are the primary factors that contribute to the increase in the concentration of major ions in groundwater.

**Fig. 3:**
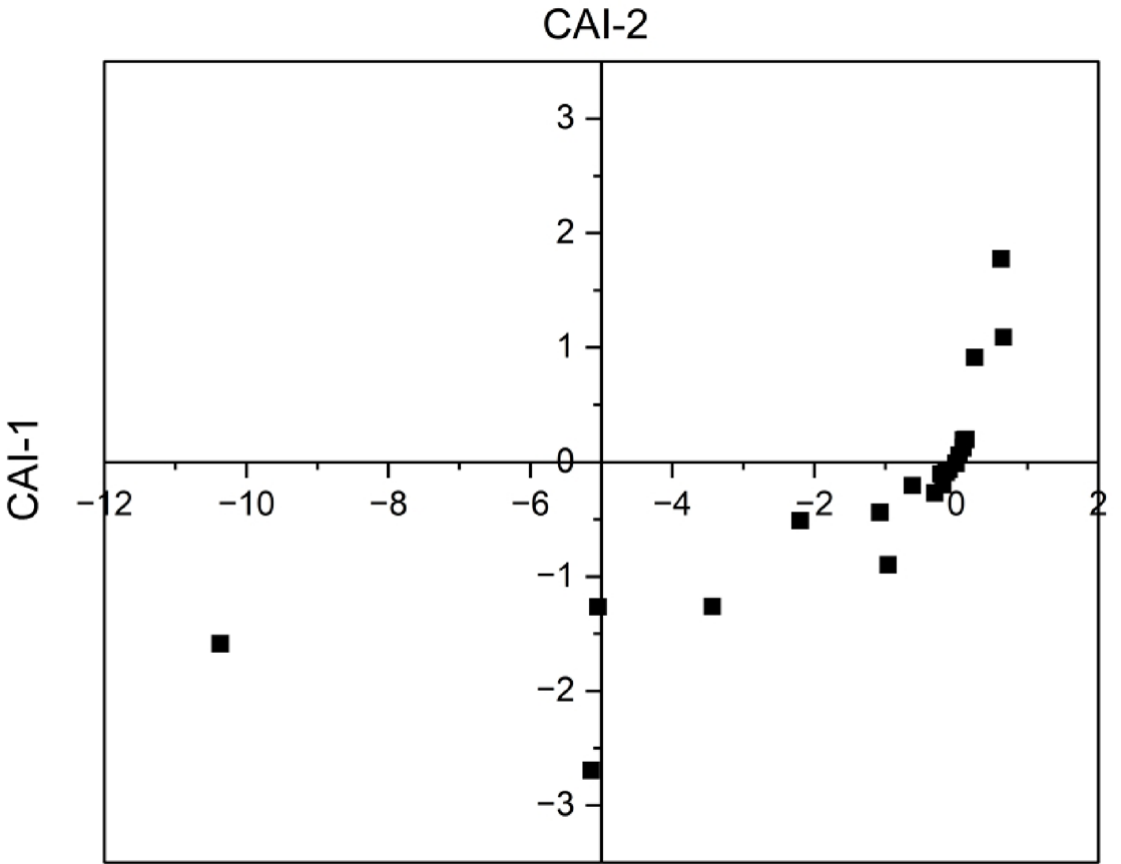
Ion exchange mechanism of groundwater based on chloroalkaline indices.

Understanding the changes that occur in the chemical structure of groundwater during its subterranean transit is crucial. CAI concentrations as shown in Fig. 3 which indicates that 35% of sampling sites of water samples exhibit positive ratios and 65% of sampling sites of samples correspond to negative ratios, illustrating the kind of base exchange. Positive results indicate the absence of base exchange and the process is classified as a cation-anion exchange reaction. The negative concentrations of the ratio represent base exchange between Na^+^ and K^+^ in water with Ca^2+^ and Mg^2+^ in the rocks.

### Suitability of groundwater for irrigation application

The groundwater quality in the area under investigation was evaluated in order to determine whether or not it is suitable for potable consumption, irrigation, and industrial applications.

#### Sodium percent (%Na) and sodium adsorption ratio (SAR)

The groundwater quality in the area under investigation was evaluated in order to determine whether or not it is suitable for potable consumption, irrigation, and industrial applications.

**Table 2:** Classification of water using sodium percent (%Na).

%Na	Class of water	Samples number	%
< 20	Excellent	20	5
20–40	Good	3, 4, 5, 6, 7	25
40–60	Permissible	6,7, 12, 13	20
60–80	Doubtful	1, 2, 4, 5, 13, 15, 17, 18,19	45
> 80	Unsuitable	16	5

Na% is a prominent indicator that is used to evaluate the degree to which it is suitable for irrigational operations. The recorded sodium percent in the investigated area varies from 16 to 88, with a mean of 54. With the exception of a few samples, the majority of groundwater samples are not appropriate for irrigation based on their sodium content shown in Table 2. The increased Na% was attributed to the prolonged residence time of water, the disintegration of minerals from the lithological structure, and the introduction of chemical fertilizers carried by the irrigation waters.

The sodium risk is commonly quantified as the Sodium Adsorption Ratio (SAR), a crucial factor in assessing the appropriateness of irrigation water. The SAR level of water used for irrigation is strongly relevant to the degree of sodium absorption by the soils. This metric measures the ratio of sodium ions to calcium and magnesium ions in a given sample. As shown in Table 3, the SAR ranged from 1.77 to 11.93 with mean value of 7.93. Table 3 summarizes the categorization of samples of groundwater collected from the research area based on SAR. All samples exhibit a SAR value below 18, indicating excellent to good suitability for irrigation.

**Table 3:** SAR based water classification in the study area.

Class of SAR	SAR range	Quality remark	No. of samples	% of samples
S_1_	< 10	Excellent	13	65
S_2_	10 to 18	Good	07	35
S_3_	18 to 26	Permissible	0	0
S_4_	> 26	Doubtful	0	0

**Table 4:** EC based types of water quality in the study area.

Classification of EC	EC (µs/cm) values	Quality remark	No. of samples	% of Water samples
C_1_	< 250	Excellent	0	0
C_2_	250 to 750	Good	0	0
C_3_	750 to 2250	Permissible	7	35
C_4_	> 2250	Doubtful	13	65

The salinity risk, as measured by EC, is the primary water quality constraint on the productivity of crops. To facilitate evaluation and categorization, the level of salts that are soluble (salinity risk) in water used for irrigation can be quantified using electrical conductivity. Table 4 displays the categorization of groundwater according to the risk of salinity. Using the analysis of the EC concentration, it was determined that only 35% collected samples fall in permissible category were appropriate to use for irrigation and soils needs with restricted drainage, while 65% water samples were not use for irrigation applications. The occurrence of saline soil is caused by high amounts of EC in water, whereas alkaline soil is caused by high levels of sodium in water.

#### Wilcox and USSL diagram

**Fig. 4:**
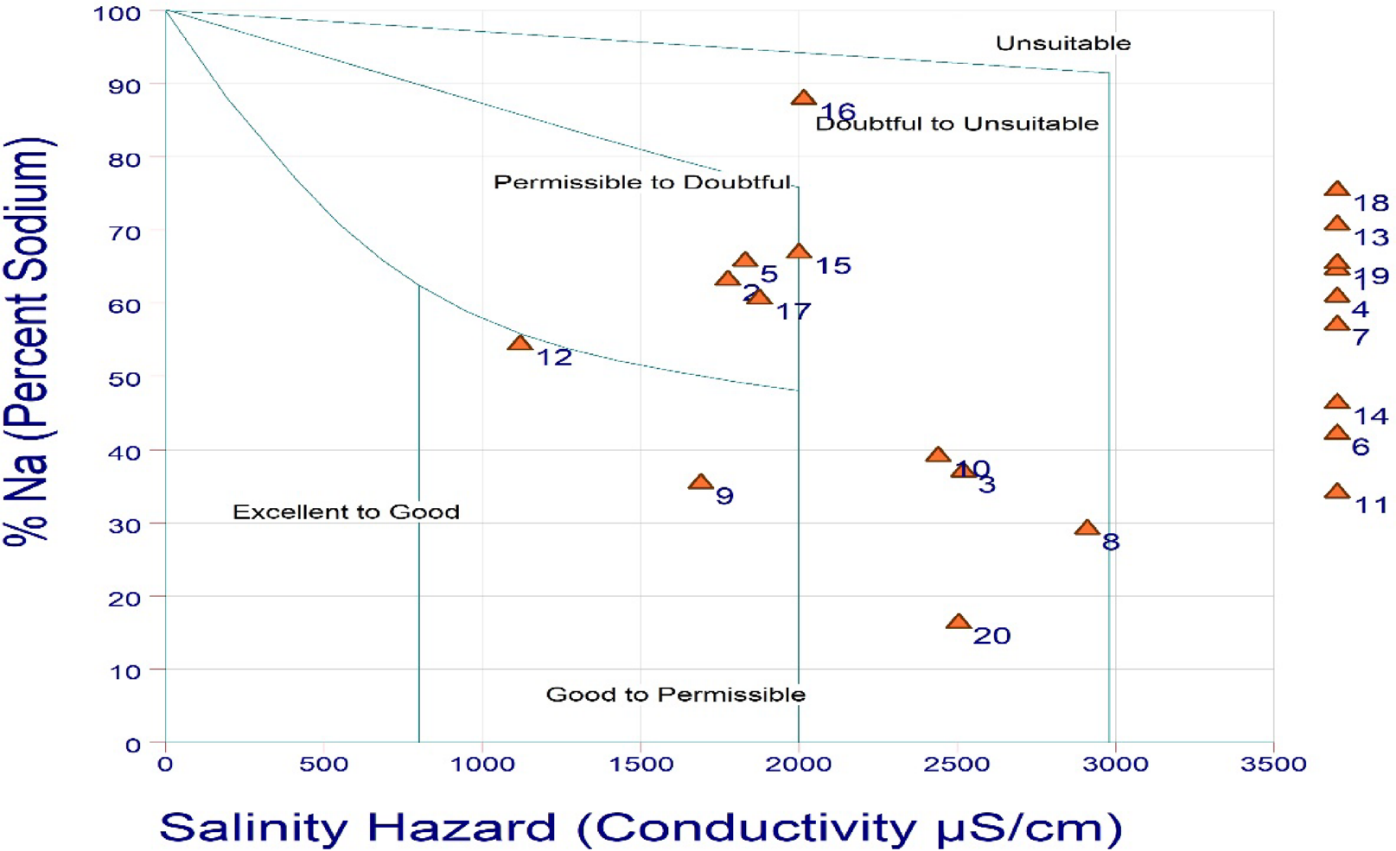
Groundwater quality classification based on Wilcox diagram.

When determining whether or not groundwater is suitable for irrigation, Wilcox (1955) utilized the percentage of sodium as well as the specific conductivity test^40^. Wilcox (1955) indicates that the water quality diagram for agricultural and domestic use categorizes 10% of water sampling sites as good to permissible, 20% sampling sites as permissible to doubtful, 25% of water sampling sites doubtful to unsuitable, and 45% of groundwater sampling sites as unsuitable category (Fig. 4). High concentrations of sodium in irrigation water result in the absorption of sodium ions by particles of clay, thereby eliminating magnesium and calcium ions.

**Fig. 5:**
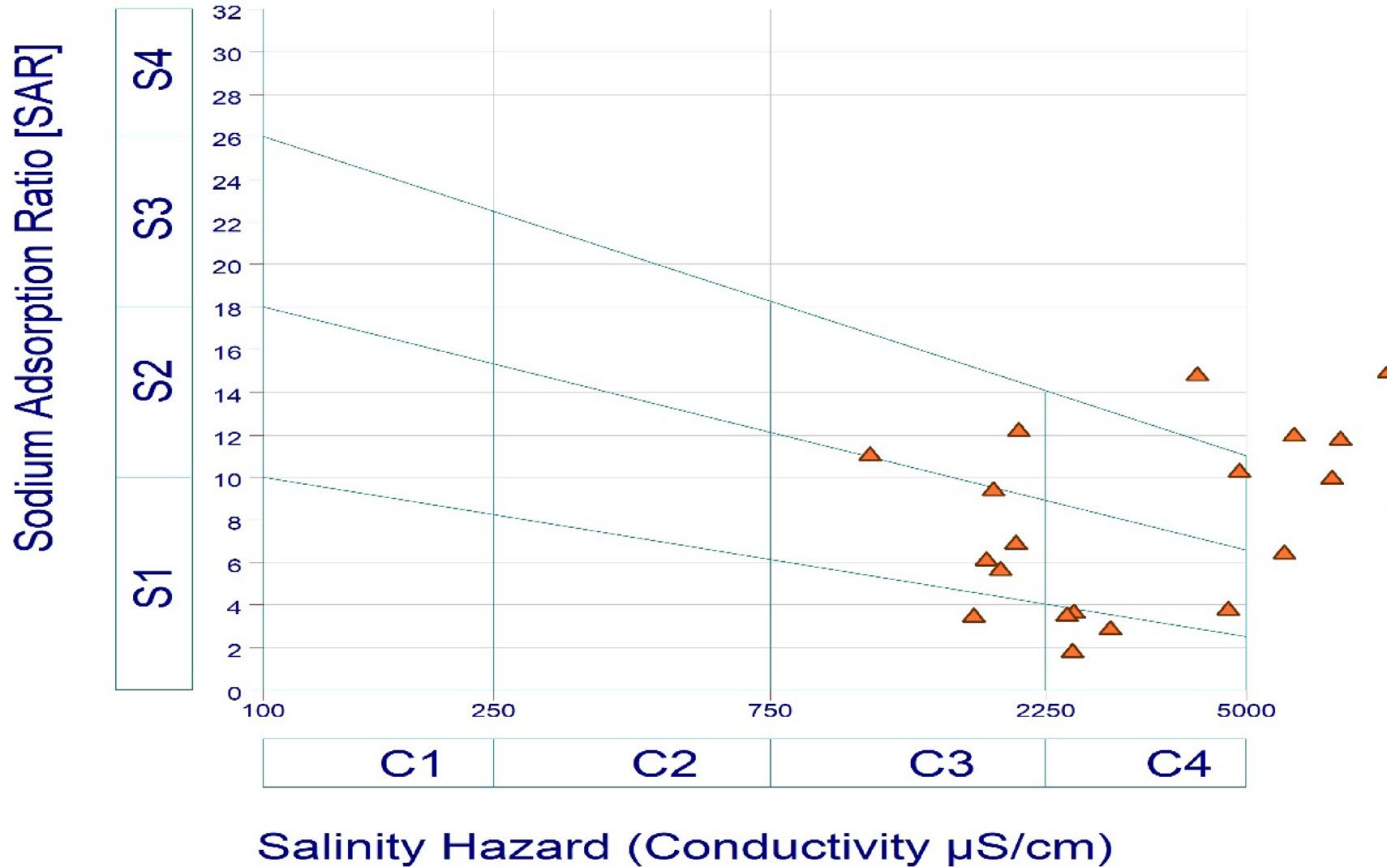
USSL based water quality classification.

In the USSL graph, conductivity (EC) is regarded as a salinity hazard, while sodium adsorption ratio (SAR) is considered an alkalinity hazard. The SAR and EC measurements for samples of groundwater from the research area were illustrated in the USSL pictorial diagram (Fig. 5) for water used for irrigation^41^. The 20% of water sampling sites are classified as C4S1 quality, exhibiting a significant salinity hazard and low sodium content. The 25% and 30% of sampling sites are as classified as C4S2 and C4S4 categories indicating very high salinity to medium sodium and very high salinity to very high sodium content. The excessive salinity levels could originate from geological structures in the region, where rainfall leaches salt from the soil and introduces both domestic waste and fertilizers into the groundwater. The assessment of samples of groundwater concerning salinity and sodium hazard indicates that high sodium ion concentrations at some locations may result in detrimental levels of exchangeable sodium levels in the soil. According to the USSL diagram results, the majority of the groundwater sampling sites are not satisfactory for irrigation across nearly all soil types.

### Computation of multivariate statistical techniques

Multivariate statistical techniques (MSTs), such as cluster analysis and discriminant analysis, are frequently employed in environmental studies to assess analytical data for identifying possible factors^60,61^. Multivariate analysis was conducted employing SPSS 22 for Windows in our study.

#### Cluster analysis

**Fig. 6:**
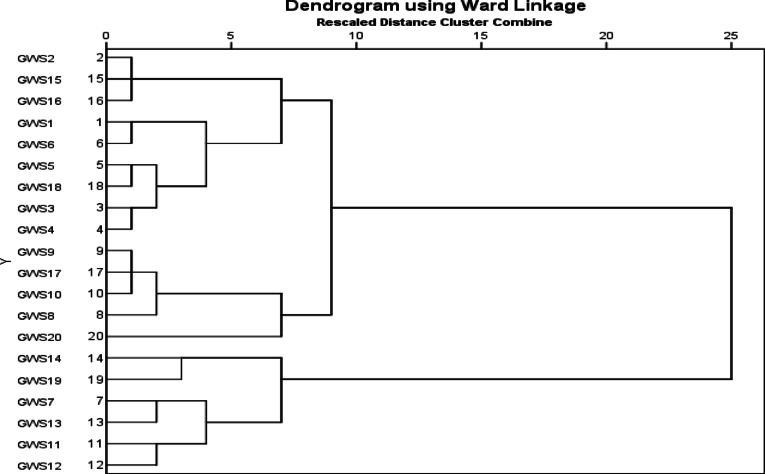
Dendrogram illustrating the HCA of the analyzed groundwater sampling locations.

Clustering analysis consists of various multivariate techniques employed to identify authentic data clusters^62^. The HCA analysis employed in the present investigation is an extremely commonly utilized method for evaluating the quality of water data. Cluster analysis was utilized to estimate hydrochemical classifications within the groundwater information. CA for groundwater datasets generated a dendrogram categorizing the 20 different locations for sampling into three statistically significant clusters (cluster 1, cluster 2, and cluster 3) as shown in Fig. 6. Cluster 1 comprises sampling points GWS1 to GWS6, GWS15, GWS16 and GWS18. Cluster 2 comprises the sample sites GWS7, GWS11 to GWS14 and GWS19 while Cluster 3 includes the sampling sites GWS8 to GWS10, GWS17 and GWS20. Parameters within the exact same cluster had been likely derived from a common source. The categorization of sampling points in cluster 2 exhibited significantly high range of contamination in comparison to cluster 2. Although parameter levels in cluster 1 and cluster 3 are relatively lower, certain parameters still surpassed the acceptable limits. This Cluster 2 location is sealed off from contamination of the groundwater due to dissolution of minerals in aquifer, agricultural fertilizers and sewage leaching. Mean concentration of parameters exhibiting a standard deviation exceeding 300. Cluster 1and cluster 3 consist sites generally exhibit reduced groundwater contamination from runoff from agricultural operations and surface. The CA method effectively provides precise characterization of groundwater throughout the region.

#### Discriminant analysis

DA has been applied to determine the key parameters affecting the fluctuations in the quality of groundwater. It was conducted utilizing characteristics of water quality as predictors for classification within water quality groups derived from cluster analysis^4^.

**Table 5:** Box’s M test of covariance matrices.

Box’s M	F Approx.	Df1	Df2	Sig
11.085	1.498	6	2300.348	0.175

**Table 6:** Wilk’s lambda and chi-square test for the DA.

Function	Canonical correlation	Eigen value	Cumulative %	Wilk’s lambda	Chi-square	Df	Sig
1	0.945	8.345	94.6	0.073	43.290	4	0.000
2	0.568	0.475	100	0.678	6.415	1	0.011

The Box’s M test and statistical description of the DA is presented in Tables 5 and 6; Wilks’ lambda and Chi-square ranges 0.073 to 0.678 and 43.290 to 6.415 respectively were employed to assess the statistical importance of the discriminant function (Table 6). Box’s tests value 11.085 revealed a significant difference in the covariance matrix of the dependent variables across groups (*p* < 0.05). The relatively small Wilks’ lambda and an increased Chi-square index signify the substantial prominence of the discriminant function.

**Table 7:** Classification function coefficient for DA.

	Cluster		
	1.00	2.00	3.00
TDS	0.003	0.005	0.001
Cl^−^	0.000	0.022	0.007
(Constant)	− 4.011	− 31.061	− 3.374

**Fig. 7:**
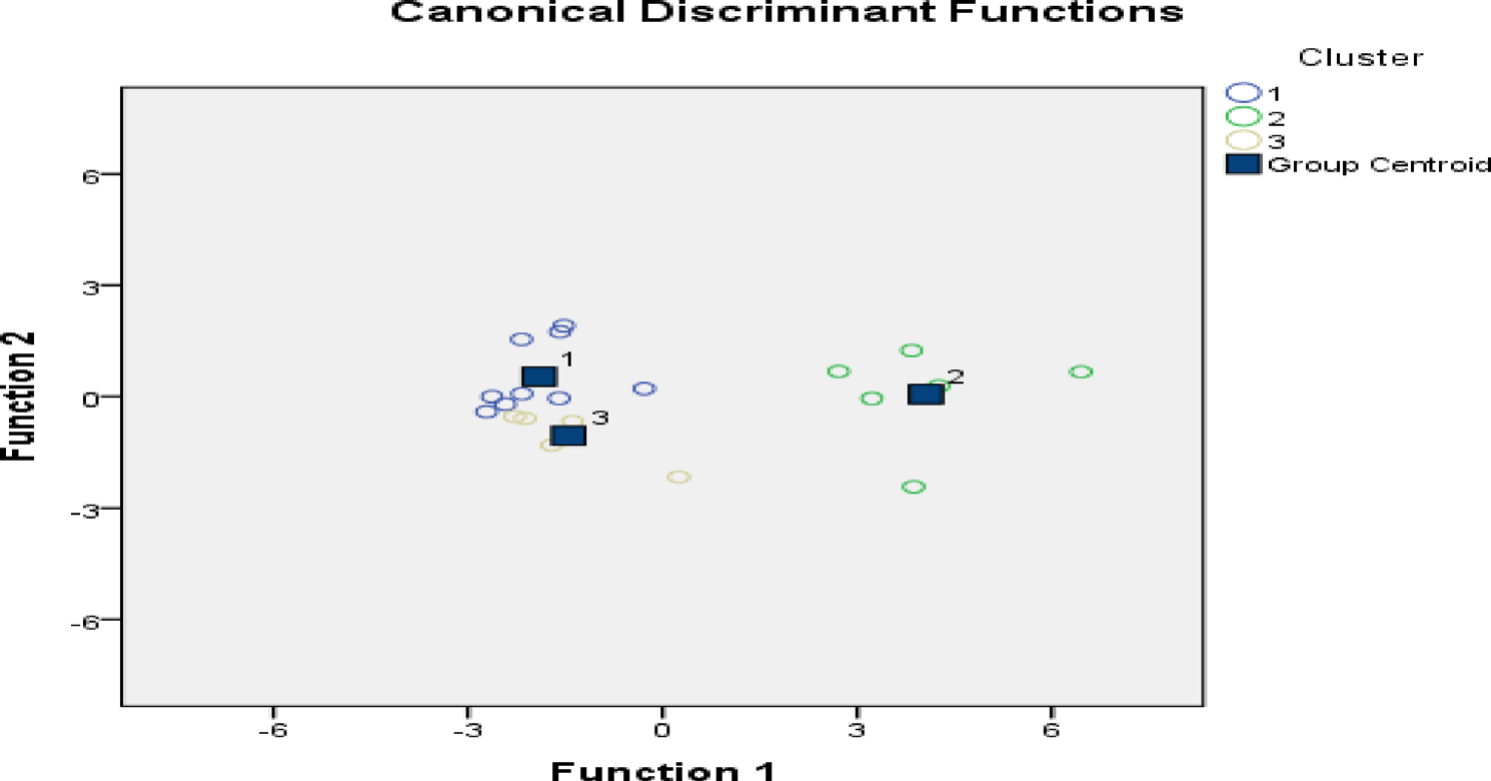
Summary of conical discriminant functions.

The discriminant function coefficients produced in the DA for two major variables (TDS and Cl^−^) are listed in Table 7. The amount of DF coefficients assesses the efficacy of each of these components; an increased relative coefficient value signifies that the parameter occupies a significant role in discriminant analysis. The canonical functions coefficient is shown in Fig. 7. All predicted probabilities of the computational classification above 100%, as indicated in Table 8.

**Table 8:** Classification results based on clustering for DA.

		Cluster	Predicted group membership			Total
			1.0	2.0	3.0	
Original	Count	1	9	0	0	9
		2	0	6	0	6
		3	0	0	5	5
	%	1	100.0	0.0	0.0	100.0
		2	0.0	100.0	0.0	100.0
		3	0.0	0.0	100.0	100.0

#### Computation of water quality index

**Fig. 8:**
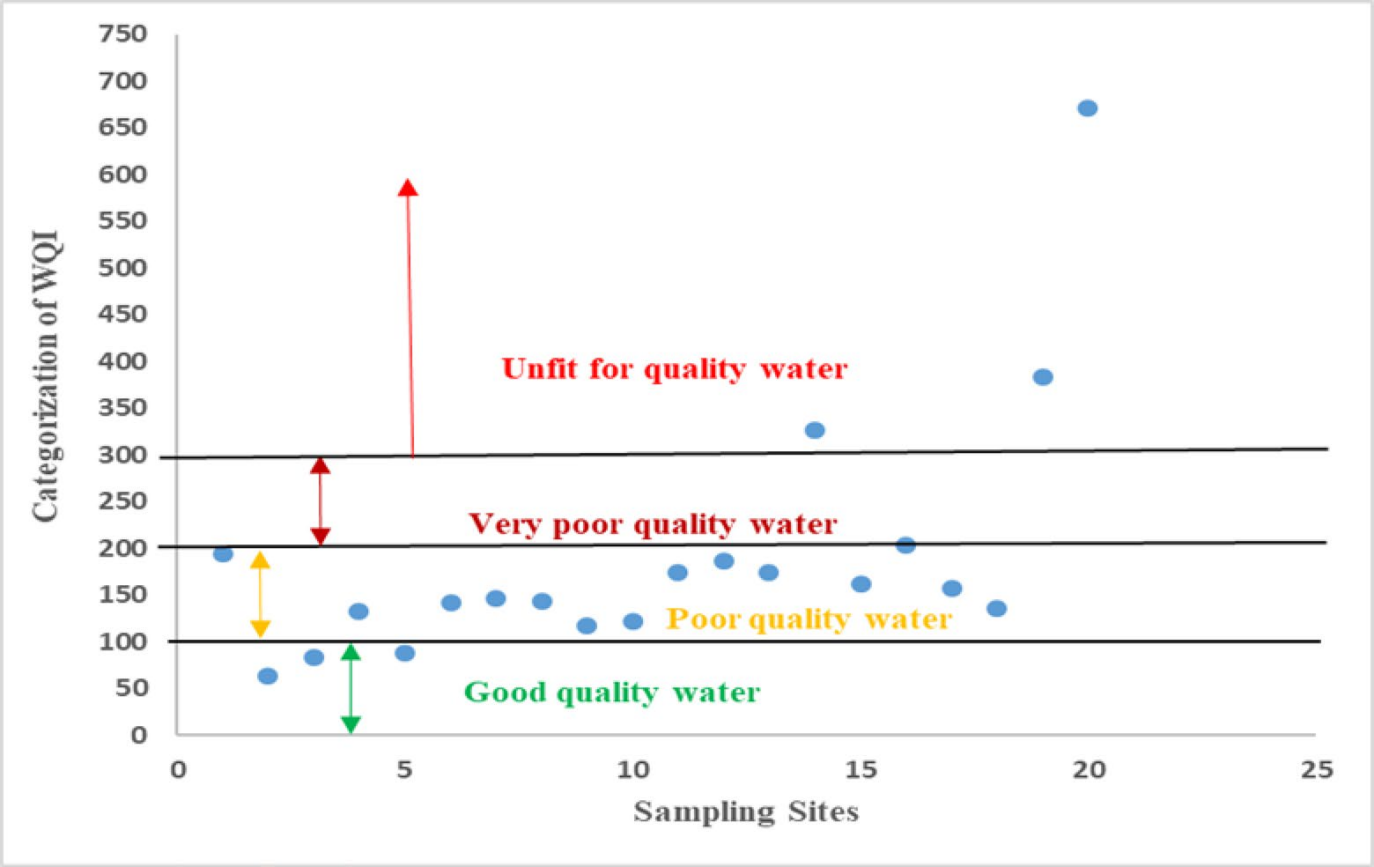
Categorization of groundwater based on WQI.

Water quality classifications were established according to the Arithmetic Water Quality Index (WQI). The calculated WQI 64.17 to 671.51 with the average value of 190.49. Table 9 categorizes the WQI range and water types, revealing that most samples surpass the allowable levels established by BIS (2012) and WHO (2011) standards^31,32^. Among sampling sites none of the samples have been found in excellent quality, 7.14% have been found as good quality, 3.57% sampling locations have been categorize in poor quality. Whereas, 15% of water sampling sites have been classified in water not suitable for drinking uses category as shown in Fig. 8. This may result from efficient ion leaching, excessive groundwater extraction, direct effluent discharge, and agricultural influences^63–67^.

**Table 9:** Classification of water quality based on WQI.

Range	Types of water	% of Samples
< 50	Excellent quality	0
50–100	Good quality	15
100–200	Poor quality	65
200–300	Very poor quality	5
> 300	Unsafe for drinking	15

### Limitation and recommendations

Present study is limited to a specific season. Seasonal changes, including variations in precipitation patterns and hydrological conditions, can influence groundwater quality parameters. Since this study is based on sampling in single pre monsoon season (April and May 2024) thus seasonal variations cannot be represented. It is recommended to the decision-making authorities that at present time various portable instruments and automated sensors are used to measure and evaluate the physico-chemical properties of groundwater in real time. Automated sensors are generally installed in monitoring wells. After measuring these sensors transmit data remotely using wireless communication. Various other methods are also available for ground water quality monitoring like Conducting tests like pump tests and borehole tests to assess the permeability and water flow characteristics of the groundwater. These are useful in determining how contaminants may spread in the aquifer. Chemical tracers are added to the water to help trace flow and determine the movement of contaminants within the groundwater system. Monitoring plan should be designed based on local geology, land use, and expected contaminant sources.

## Conclusion

The present investigation emphasizes the significance of hydrogeochemical approaches such as MSTs, hydrochemical facies irrigation indices and WQI, in elucidating the origins and development of groundwater in the Mathura region, as well as in evaluating its appropriateness for use by people and irrigation purposes. The present novel assessment reveals significant insights for both public health and agricultural sustainability. The analysis identifies geographic trends and pollution clusters while facilitating informed decision-making for groundwater resource management in a region under enhanced anthropogenic and climatic challenges. This study illustrated the significance of multivariate statistical analysis including CA and DA in quality of groundwater research. Fundamental statistics indicated that the minority of variables above the predetermined desirable levels, while a majority also exceeded the permissible limits. HCA effectively classified the 20 different locations for sampling into three primary clusters based on contamination concentration in the study areas. This aids in identifying problematic areas requiring targeted corrective measures. DA effectively identified several indicator characteristics that contributed to notable geographic variations in the state of groundwater within the scope of investigation area. DA of the two variables (TDS and Cl^−^) attained a conformity rate of 100%, enabling the reduction of information. Hydrochemical diagrams, including the Piper plot and ion mechanism utilizing CAI indices, indicated the predominant groundwater facies. The SAR and %Na was employed to evaluate the irrigation suitability of groundwater. Based on the USSL and Wilcox diagrams, most groundwater samples are deemed incompatible with irrigation practices. Moreover, irrigation indices like Sodium Adsorption Ratio (SAR) demonstrated that certain groundwater samples are appropriate for agricultural application, whilst others provide moderate to severe threats to soil integrity and crop yield. According to the WQI, majority of the groundwater sampling sites were found in category of poor water quality for drinking purposes, needing rapid management intervention. The quality of groundwater in the investigated regions has been influenced by both naturally occurring hydrochemical mechanisms such as minerals dissolution and weathering of rock formations and anthropogenic contamination sources including excessive development of urbanization, leaching of soil and waste, runoff high use of fertilizers, and sewage infiltration. The integrated methods demonstrated robustness and efficiency in assessing and analyzing groundwater quality characteristics in the study region. This integrated review underlines the urgent need for targeted groundwater monitoring, pollution source control, and sustainable water resource management measures in the region. This research advised for the implementation of a quality of groundwater monitoring system that ensures the sustainability of the supply of water. Furthermore, regular evaluation and sustainable management of groundwater resources are imperative for their long-term utilization, necessitating the preservation of quality and the implementation of optimal methods, especially in agriculture and the planning of land uses. The findings provide a scientific basis for decision-making and policy formation aimed at protecting both human health and agricultural sustainability. The investigation has a limitation in that the evaluation was conducted primarily for Spatial variation seasons; nevertheless, the quality of the water may vary during different seasons. These data can also offer crucial insights into the water quality utilized for of drinking and agriculture operations and promoting sustainable and effective management of water resources in the region and nearby locations. Additionally, a further study on contamination of groundwater from point and non-point sources could be addressed to develop sustainable management approaches. This study conducted for a specific season of the year.

## Electronic supplementary material

Below is the link to the electronic supplementary material.

Supplementary Material 1.

## Data Availability

The datasets used and/or analysed during the current study available from the corresponding author on reasonable request.
