# Supplementary material for: Integrated evaluation of groundwater hydrochemistry using multivariate statistics and irrigation-based water quality indices
Source: Sci Rep. 2025 Jul 10;15:24923. doi: 10.1038/s41598-025-09874-3 (PMC12246167; doi:10.1038/s41598-025-09874-3)
Supplement: Supplementary file 1 — Supplementary Material 1. [file 41598_2025_9874_MOESM1_ESM.pdf]

SPRINGER NATURE

Change of authorship request form - Journals (pre-acceptance)

Important information. Please read.

- This form should be used by authors to request any change in authorship (adding/deleting authors) including changes in corresponding authors. This form should not be used for name changes. Please fully complete all sections. Use black ink and block capitals and provide each author's full name with the given name first followed by the family name.

- By signing this declaration, all authors guarantee that the order of the authors are in accordance with their scientific contribution, if applicable as different conventions apply per discipline, and that only authors have been added who made a meaningful contribution to the work.

- Please note, in author collaborations where there is formal agreement for representing the collaboration, it is sufficient for the representative or legal guarantor (usually the corresponding author) to complete and sign the Authorship Change Form on behalf of all authors, **next to the added/removed author(s)**. **(Complete Section 3, followed by Section 6.)**
  In author collaborations where there is no formal agreement for representing the collaboration and **there are more than 10 authors**, one may sign for all, provided the signer appends correspondence that attests that each of the authors have agreed to the change **and the added/removed authors sign the form**. **(Complete Section 3, followed by Section 6.)**

- Please note, we cannot investigate or mediate any authorship disputes. If you are unable to obtain agreement from all authors (including those who you wish to be removed) you must refer the matter to your institution(s) for investigation. Please inform us if you need to do this.

- If you are not able to return a fully completed form within **30 days** of the date that it was sent to the author requesting the change, we may have to withdraw your manuscript. We cannot publish manuscripts where authorship has not been agreed by all authors (including those who have been removed).

- Incomplete forms will be rejected.

- Please return/upload this form, fully completed, to the Journals Editorial Office. The Journal and/or Publisher will consider the information you have provided to decide whether to approve the proposed change in authorship. We may decide to contact your institution for more information or undertake a further investigation, if appropriate, before making a final decision.

SPRINGER NATURE Springer Nature is one of the world's leading global research, educational and professional publishers, created in May 2015 through the combination of Nature Publishing Group, Palgrave Macmillan, Macmillan Education and Springer Science+Business Media.

SPRINGER NATURE

Change of authorship request form - Journals (pre-acceptance)

Section 1: Please provide the current title of manuscript

Manuscript ID no.: a573a287-80e0-468f-b506-dfc4ab24236b v2.0

Title: **Integrated Evaluation of Groundwater Hydrochemistry Using Multivariate Statistics and Irrigation-Based Water Quality Indices**

Section 2: Please provide the previous authorship, in the order shown on the manuscript before the changes were introduced. Please indicate the corresponding author by adding (CA) behind the name.

	First name(s)	Family name	ORCID or SCOPUS id, if available
1st author	Raisul	Islam	
2nd author	Vinod Kumar	Kushwah	
3rd author	Nakul	Gupta	
4th author	Ashish	Kumar	
5th author	Rajesh	Goyal	
6th author	Parveen	Berwal	
7th author	Faisal M.	Alfaisal	
8th author	Ali	Majdi	
9th author	Osamah J.	Alsareji	
10th author			

Please use an additional sheet if there are more than 10 authors.

SPRINGER NATURE Springer Nature is one of the world's leading global research, educational and professional publishers, created in May 2015 through the combination of Nature Publishing Group, Palgrave Macmillan, Macmillan Education and Springer Science+Business Media.

SPRINGER NATURE

Change of authorship request form - Journals (pre-acceptance)

Section 3: Please provide a justification for change. Please use this section to explain your reasons for changing the authorship of your manuscript, e.g. what necessitated the change in authorship? Please refer to the (journal) policy pages for more information about authorship. Please explain why omitted authors were not originally included and/or why authors were removed on the submitted manuscript.

Dr. **Majed Alsubih** has been added as a co-author in the revised manuscript due to his significant contributions during the revision phase. He was actively involved in refining the manuscript's structure and scientific clarity, particularly in aligning the study with irrigation water quality frameworks. His expert input also contributed to addressing reviewers' comments and enhancing the overall methodological robustness and coherence of the study.

Section 4: Proposed new authorship. Please provide your new authorship list in the order you would like it to appear on the manuscript. Please indicate the corresponding author by adding (CA) behind the name. <u>If the Corresponding Author has changed, please indicate the reason under section 3</u>.

	First name(s)	Family name (this name will appear in full on the final publication and will be searchable in various abstract and indexing databases)	Affiliated institute	E-mail address
1st author	Raisul	Islam	Civil Engineering Department, GLA University, Mathura, Uttar Pradesh (India)	*raisul.islam@gla.ac.in*
2nd author	Vinod Kumar	Kushwah	Civil Engineering Department, GLA University, Mathura, Uttar Pradesh (India)	*vinnikushwah@gmail.com*
3rd author	Nakul	Gupta	*NICMAR Institute of Construction Management and Research, NICMAR, Delhi-NCR, Bahadurgarh, India-124507*	*nakul030588@gmail.com*
4th author	Ashish	Kumar	*Division of Research and Development, Lovely Professional University, Phagwara, India*	*akul030588@gmail.com*
5th author	Rajesh	Goyal	*NICMAR Institute of Construction Management and Research, NICMAR, Delhi-NCR, Bahadurgarh, India-124507*	*rgoyal@nicmar.ac.in*
6th author	Parveen	Berwal	*Civil Engineering Department. Galgotias College of Engineering & Technology, Greater Noida, 201310, India*	parvveenn@gmail.com
7th author	Faisal M.	Alfaisal	*Department of Civil Engineering, College of Engineering, King Saud University, Riyadh 11421, Saudi Arabia*	*falfaisal@ksu.edu.sa*
8th author	Ali	Majdi	*Department of Buildings and Construction*	. *alimajdi@uomus.edu.iq*

SPRINGER NATURE Springer Nature is one of the world's leading global research, educational and professional publishers, created in May 2015 through the combination of Nature Publishing Group, Palgrave Macmillan, Macmillan Education and Springer Science+Business Media.

			Techniques Engineering, College of Engineering, Al-Mustaqbal University, 51001 Hillah, Babylon , Iraq	
9th author	Osamah J.	Alsareji	*Sustainability Solutions Research Lab, Faculty of Engineering, University of Pannonia, Egyetem Str. 10, Veszprém H, 8200, Hungary*	osamah.al-sareji@phd.uni-pannon.hu
10th author	Majed	Alsubih	*Civil Engineering Department, College of Engineering, King Khalid University, Abha 61421, Saudi*	malsubih@kku.edu.sa

Please use an additional sheet if there are more than 10 authors.

SPRINGER NATURE

Change of authorship request form - Journals (pre-acceptance)

Section 5: Author contribution, Acknowledgement and Disclosures. Please use this section to provide a new disclosure statement and, if appropriate, acknowledge any contributors who have been removed as authors and ensure you state what contribution any new authors made (if applicable per the journal or book (series) policy). Please ensure these are updated in your manuscript - after approval of the change(s) - as our production department will not transfer the information in this form to your manuscript.

New acknowledgements:

New Disclosures (financial and non-financial interests, funding):

New Author Contributions statement (if applicable per the journal policy):

"R.I.:; writing—original draft; writing—review and editing; V.K.K.: writing—original draft; writing—review and editing; N.G.: writing—original draft; writing—review and editing; A.K.: writing—original draft; writing—review and editing; R.G.: writing—original draft; writing—review and editing P.B: writing—original draft; writing—review and editing; F.M.A.: writing—original draft; writing—review and editing, A.M.: writing—original draft; writing—review and editing, O.J.A.: writing—original draft; writing—review and editing. M.A.:writing—review and editing, All authors have read and agreed to the published version of the manuscript"

SPRINGER NATURE Springer Nature is one of the world's leading global research, educational and professional publishers, created in May 2015 through the combination of Nature Publishing Group, Palgrave Macmillan, Macmillan Education and Springer Science+Business Media.

SPRINGER NATURE

Change of authorship request form - Journals (pre-acceptance)

State 'Not applicable' if there are no new authors.

Change of authorship request form - Journals (pre-acceptance)

Section 6: Declaration of agreement. *All* authors, unchanged, new and removed *must* sign this declaration.
(NB: Please print the form, (docu)-sign and return/upload a scanned copy. Please note that signatures that have been inserted as an image file are acceptable as long as it is handwritten. Typed names in the signature box are unacceptable.) *** Please delete as appropriate. Delete all of the bold if you were on the original authorship list and are remaining as an author.**

	First name	Family name		Signature	Date
1st author	Raisul	Islam	I agree to the proposed new authorship shown in section 4 **/and the addition/removal*of my name to the authorship list** /and the proposed change in corresponding author		15/5/2025
2nd author	Vinod Kumar	Kushwah	I agree to the proposed new authorship shown in section 4 **/and the addition/removal*of my name to the authorship list** /and the proposed change in corresponding author		15/5/2025
3rd author	Nakul	Gupta	I agree to the proposed new authorship shown in section 4 **/and the addition/removal*of my name to the authorship list** /and the proposed change in corresponding author		15/5/2025
4th authors	Ashish	Kumar	I agree to the proposed new authorship shown in section 4 **/and the addition/removal*of my name to the authorship list** /and the proposed change in corresponding author		15/5/2025

SPRINGER NATURE Springer Nature is one of the world's leading global research, educational and professional publishers, created in May 2015 through the combination of Nature Publishing Group, Palgrave Macmillan, Macmillan Education and Springer Science+Business Media.

SPRINGER NATURE

Change of authorship request form - Journals (pre-acceptance)

5th author	Rajesh	Goyal	I agree to the proposed new authorship shown in section 4 /**and the addition/removal*of my name to the authorship list** /and the proposed change in corresponding author	Rajesh	15/5/2025
6th author	Parveen	Berwal	I agree to the proposed new authorship shown in section 4 /**and the addition/removal*of my name to the authorship list** /and the proposed change in corresponding author	PB	15/5/2025
7th author	Faisal M.	Alfaisal	I agree to the proposed new authorship shown in section 4 /**and the addition/removal*of my name to the authorship list** /and the proposed change in corresponding author	Faisal	15/5/2025

SPRINGER NATURE Springer Nature is one of the world's leading global research, educational and professional publishers, created in May 2015 through the combination of Nature Publishing Group, Palgrave Macmillan, Macmillan Education and Springer Science+Business Media.

SPRINGER NATURE

Change of authorship request form - Journals (pre-acceptance)

	First name	Family name		Signature	Date
8th author	Ali	Majdi	I agree to the proposed new authorship shown in section 4 /**and the addition/removal*of my name to the authorship list** /and the proposed change in corresponding author	Ali Majdi	15/5/2025
9th author	Osamah J.	Alsareji	I agree to the proposed new authorship shown in section 4 /**and the addition/removal*of my name to the authorship list** /and the proposed change in corresponding author	Osamah Alsareji	15/5/2025
10th author	Majed	Alsubih	I agree to the proposed new authorship shown in section 4 /**and the addition/removal*of my name to the authorship list** /and the proposed change in corresponding author	[illegible]	15/5/2025

Please use an additional sheet if there are more than 10 authors.

In case of author collaborations with formal agreement:

	Name of consortium/consortia	First name	Family name		Signature	Date
Representative/legal guarantor				I agree to the proposed new authorship shown in section 4 /**and the addition/removal*of my name to the authorship list** /and the proposed change in corresponding author		

Both added/removed authors should complete the information in the first table under Section 6.

SPRINGER NATURE Springer Nature is one of the world's leading global research, educational and professional publishers, created in May 2015 through the combination of Nature Publishing Group, Palgrave Macmillan, Macmillan Education and Springer Science+Business Media.

---- End of form ----

SPRINGER NATURE Springer Nature is one of the world's leading global research, educational and professional publishers, created in May 2015 through the combination of Nature Publishing Group, Palgrave Macmillan, Macmillan Education and Springer Science+Business Media.
